# Policy implementation of methadone maintenance treatment and HIV infection: evidence from Hubei province, China

**DOI:** 10.1186/1747-597X-8-38

**Published:** 2013-11-05

**Authors:** Jifang Dai, Lianyi Zhao, Yuan Liang

**Affiliations:** 1 AIDS and STD Prevention and Control Center, Institute of Communicable Disease Prevention, Hubei Center for Diseases Control and Prevention, Wuhan 430079, China; 2 Department of Social Medicine and Health Management, School of Public Health, Tongji Medical College, Huazhong University of Science and Technology, Hangkong Road 13, Wuhan 430030, China

**Keywords:** Human immunodeficiency virus, Methadone maintenance treatment, Drug abusers, China

## Abstract

To view methadone maintenance treatment (MMT) globally, it is necessary to accumulate data on MMT policy implementation under different health service systems. The aim of the current study is to provide empirical evidence about policy implementation of MMT and HIV infection control, as well as recommendations for improvement of MMT in the future. Based on China’s national policy framework of MMT, policy implementation of MMT in Hubei province has two objectives: 1) to create linkages between health and public security, and 2) to provide integrated services for management of drug abusers. From 2007 to 2011, following the establishment of MMT clinics that provide methadone as well as HIV prevention services, the proportion of HIV infection among drug abusers decreased relatively quickly (12.12% → 5.77% → 5.19% → 2.39% → 2.04%). However, high drop-out rate and poor information management have been identified as particular problems which now need to be addressed. Furthermore, client drop-out from MMT programs may reflect social issues the clients encounter, and consequently, sustainable MMT development requires incorporation of social measures that help MMT clients return to society without discrimination, especially through family cooperation and employment opportunities.

## Introduction

Addiction to heroin and other opioids poses serious problems for individuals, families, and communities as well as society as a whole. Solutions are sometimes ambiguous, difficult, and controversial [1,2]. Since its development more than 40 years ago, methadone maintenance treatment (MMT) has been adopted by an increasing number of countries to reduce the harmful effects of heroin use [3-5]. Health-related functions of MMT programs generally have two components: reducing opiate addiction, and reducing addiction-related HIV risk behaviors, thereby reducing HIV transmission [6-8].

MMT programs function differently in different countries. In Europe, the United States, Canada, and other Western countries, there are four main providers of MMT: family physicians, multidisciplinary clinics, private clinics, and prisons [8-12]. Most service delivery is through family physicians and private clinics. In large urban centers, multidisciplinary clinics are common, especially community health clinics that provide MMT along with other medical and health promotion services. In addition to these three models of MMT service, MMT is offered in provincial prisons. The clinical use of methadone differs widely across countries. When it is used, it is commonly reported to have a high dropout rate. Taking an international perspective, the reasons for methadone treatment dropout include waiting lists, lack of money or health insurance, trepidation regarding the social stigma and discrimination that may result from identity exposure due to requirements to possess a photo identification card, beliefs about methadone side effects, and fear of withdrawal from methadone during incarceration [8,13-15]. Typical solutions to methadone treatment dropout include providing public financial support, waiving photo identification requirements, permitting time-limited treatment with the option to extend such treatment upon request, and working with corrections agencies to ensure continued methadone treatment [12,16,17]. It is worth noting that there are few studies of the impact of these solutions on treatment success in the existing literature. Further study, especially combining policy practice and comparisons among different health service systems and different regions around the world is warranted to improve the effects of MMT policy.

Owing to differences in socio-economic factors and health service systems, MMT programs in China operate differently from those in Western countries. Most MMT in China is provided by public health institutions, unlike Western countries, where MMT is typically provided by private agencies. To gain a wider understanding of the challenges to MMT implementation globally, it is necessary to accumulate data on implementation of MMT policies under different health service systems [18-20]. Although sexual transmission is the main cause of HIV infection, China is now facing an ever-growing HIV/AIDS epidemic fuelled primarily by intravenous drug use (IDU). By the end of 2005, IDU accounted for nearly half of new HIV infections [21-23]. Therefore, the analysis of MMT policy implementation in China is useful on two levels, as it can provide information on outcomes in terms of drug abuse control as well as disease prevention, and HIV prevention in particular.

The main issues addressed by the current study are 1) how to implement MMT policy in Hubei province, China, 2) effectiveness of MMT on reduction in HIV transmission, and 3) how to improve current MMT policy. The study’s aim is to provide empirical evidence about policy implementation of MMT and HIV infection control, as well as recommendations for improvement of MMT in the future.

## Case presentation

### Policy framework of MMT in China

To strengthen AIDS prevention and control activity among intravenous drug users, health advisors to the government have strongly advocated a trial MMT program in China. In February 2003, the Ministry of Health, the Ministry of Public Security, and the State Food and Drug Administration jointly issued the Temporary Scheme for Community-based Drug Maintenance Treatment for Heroin Dependents [21].

**Figure 1:**
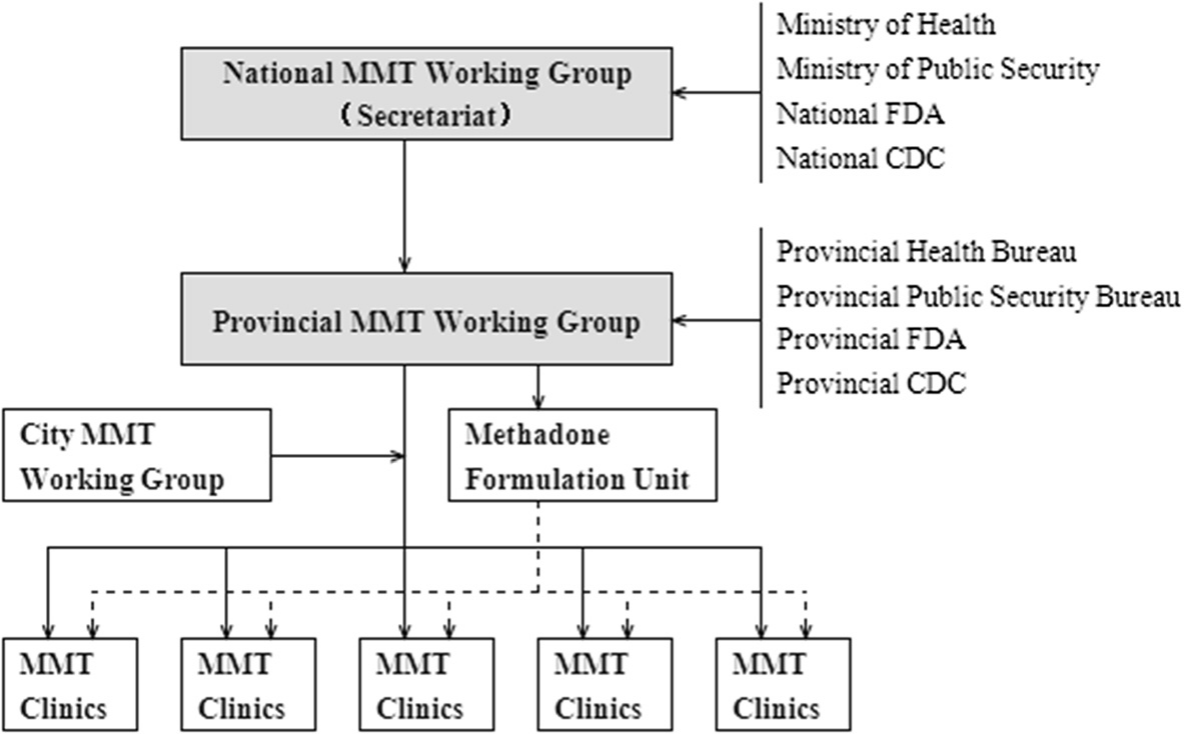
Policy framework of MMT in China.

A national working group for community-based maintenance treatment for opiate users (hereafter referred to as the National Working Group) comprises members of the Ministries of Health and Public Security, and the State Food and Drug Administration. The organizational structure of the MMT system in China includes working groups at three levels: national, provincial, and city. The MMT clinics are run by public health agencies (including public hospitals, public psychiatric hospitals, and the Chinese Center for Disease Control and Prevention), and are reviewed by the provincial working group. If clinics are approved, they are then submitted to the national working group for the record. Figure 1 shows the policy framework of MMT in China.

Because of China’s vast territory and regional differences in economic development, policy implementation of MMT is mainly completed by provincial working groups.

### Policy implementation of MMT in Hubei province, China

Hubei province is located in central China, with a population of approximately 60 million people and about 37,000 opioid addicts in 2012. However, the actual burden of opioid dependency in Hubei province may be much greater as under reporting is likely. In China, more than 30% of drug addicts use intravenous drugs, and at least 70% of IUD share syringes [19,20]. Although the drug abuse epidemic in Hubei province is moderate compared with other areas of China, drug control is particularly important because of Hubei province’s role as a traffic hub in China, as well as high population flow, relatively developed economy, and central location. Although Hubei province is considered an area with a relatively low incidence of HIV in China, by the end of 2011 a total of 7875 cases of HIV infection had reported in Hubei province.

**Figure 2:**
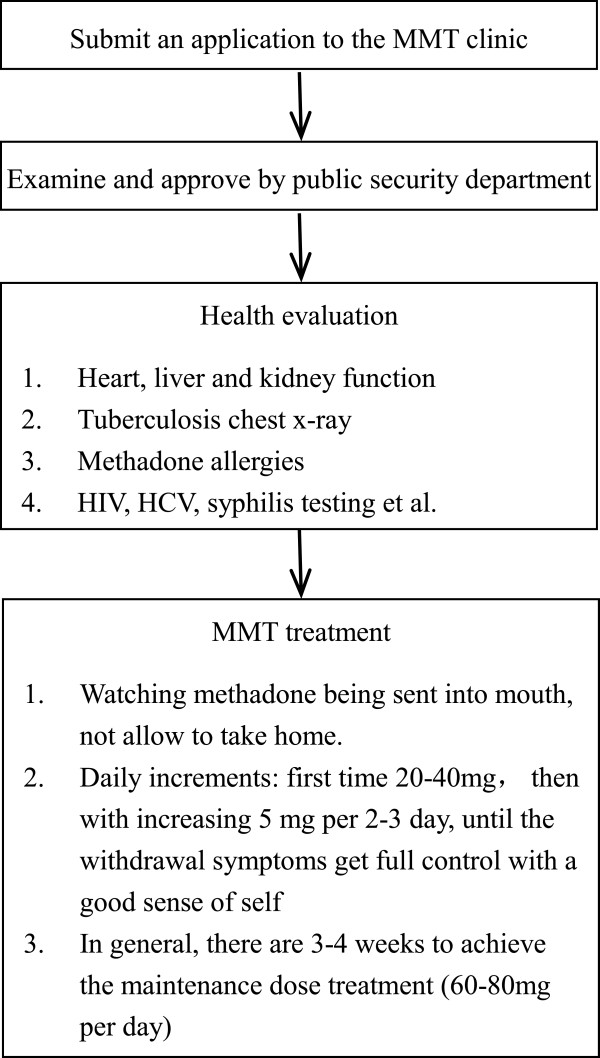
The clinical protocol in the MMT clinics in Hubei province, China.

Although the national MMT policy was introduced in 2003 in China, Hubei province started a pilot program in 2006 [24]. Figure 2 shows the clinical protocol in the MMT clinics. Following the national policy framework of MMT enacted for five years (2006–2011), policy implementation of MMT in Hubei province has two objectives: 1) to create linkages between health and private security, and 2) to provide integrated services for managing drug abusers.

### Link between health and public security in organizational management

China’s national policy framework of MMT only provides policy principles. Detailed policy implementation is organized by the provincial departments. As a result of public security threats caused by drug abuse, before 2003, drug abuse in Hubei province was mainly managed by the public security sector and executed with enforced detoxification. The detoxification model has an undeniable role in public security. However, it does not effectively solve the problem of drug-induced disease, especially HIV infection resulting from sharing syringes. Following the national policy framework of MMT in 2003, nowadays, drug abuse in Hubei province is managed by community MMT, which is led by health departments that link health and public security.

There are three aspects of the organizational management of community MMT in Hubei province. First, in terms of disease prevention and control, changing the lead department from the public security sector (namely the police department of government) to the health sector gives primary responsibility for management of drug abusers to the professional health service, which is more appropriate for the HIV prevention and control objective of MMT than the former administration management model. Second, in term of the management of drug abusers rehabilitation, community MMT is preferable to the abusers themselves who are trying to return to society and is more facilitative in promoting family and society support for them, compared with the former public security sector’s isolated drug detoxification strategy. Third, regarding public security management, information gathered by the health department (on drug abusers accessing MMT as well as disease surveillance) can be used to provide informational support for the public security department to follow and manage those drug abusers.

### Integrated services for management of drug abusers

In addition to providing methadone and needle exchanges, MMT also provides health education and counseling services related to HIV and drug abuse. It provides disease surveillance (urine morphine once per month, HIV antibodies every 6 months, HCV antibodies and syphilis testing every 12 months), condom provision, partner education, and family participation and support. The role of family and community resources cannot be underestimated in drug abuser rehabilitation. In addition, surveillance data from MMT clinics can be used not only in the health management of patients, but can also be provided for the public security department.

### Effectiveness of MMT policy implementation on HIV infection

After a pilot intervention in 2006 with 1262 drug abusers, MMT formally began, with data collected since 2007. The number of MMT clinics built in 2007 was 13, and increased by 16 in 2008, 10 in 2009, 4 in 2010, and 6 in 2011, with a total of 49 clinics currently. Table 1 shows the data from 2007–2011.

**Table 1:** Numbers of MMT clinics and patients treated in 2007–2011, Hubei province, China

**Year**	**MMT clinics**	**Patients being treated in December of this year**	**Patients treated during this year**	**Drop-out patients**	**Average daily attendance**
2007	13	4674	5918	78	4070
2008	32	11153	15335	4329	5960
2009	42	9831	17883	9151	6741
2010	46	7936	11471	3970	5242
2011	49	8115	12223	4276	4916

The initial number of MMT clinics was small, and the drop-out rate was low, perhaps because of the substantial support and coordination from local governments for those first clinics. Although the number of new entrants in 2008 and 2009 peaked, the number of drop-outs was large, with these numbers changing synchronously.

Table 2 shows HIV/AIDS infection data from MMT clinics. From 2007 to 2011, the proportion of HIV infection among drug abusers decreased relatively quickly (12.12% → 5.77% → 5.19% → 2.39% → 2.04%). Homosexual and bisexual transmission are main avenues of HIV infection. It is noteworthy that both showed an increasing tendency from 2007 to 2011, especially homosexual transmission (5.23% → 22.03% → 31.49% → 40.43% → 41.74%). In addition, it is worth noting that it is difficult for us to estimate the changes of the case number of injection drug use since the implementation of MMT in Hubei province, however, the case number of receiving MMT is increased and at the same time, the case number of infected HIV/AIDS is reduced among the case of received MMT. In other words, the reducing of HIV/AIDS caused by MMT may be occurring at the same time with the increasing of HIV/AIDS caused by sexual transmission.

**Table 2:** HIV/AIDS cases and the percent distribution in 2007–2011, Hubei province, China

**Causes of infection**	**2007**		**2008**		**2009**		**2010**		**2011**	
	**N**	**%**	**N**	**%**	**N**	**%**	**N**	**%**	**N**	**%**
Injection drug use	44	12.12	33	5.77	31	5.19	20	2.39	18	2.04
Heterosexual behavior	141	38.84	253	44.23	252	42.21	412	49.28	470	53.17
Homosexual behavior	19	5.23	126	22.03	188	31.49	338	40.43	369	41.74
Sexual and injection drug use	0	0.00	1	0.17	3	0.50	2	0.24	0	0
Blood/plasma collection	20	5.51	55	9.62	13	2.18	16	1.91	8	0.90
Blood/blood products transfusion	39	10.74	26	4.55	23	3.85	9	1.08	7	0.79
Mother-to-child transmission	8	2.20	7	1.22	3	0.50	6	0.72	5	0.57
Occupational exposure	0	0	0	0	0	0	0	0	0	0
Others	92	25.34	71	12.41	84	14.08	33	3.95	7	0.79
Total	363		572		597		836		884	

There are two aspects worth noting regarding the results presented in Table 2. The first is whether there are factors such as major public relations and marketing campaigns that are true causes of the drop in HIV cases associated with injected drug use, besides MMT. In China, policies regarding injected drug use mainly are directed towards publicity and police control. Police control is crime prevention and control of drug addicts to protect public safety. Publicity is mainly performed by the department of publicity, which is full-time and department affiliated with most government administrations (including governments at all levels and health bureaus, and education bureaus) with responsibility for publicizing the country’s major policies. Most publicity activities are relatively simple, and the main approaches are writing slogans on walls and hanging banners (with generally relatively simple messages such as “stay away from drugs, cherish life”), and developing posters, blackboards, and short films for television [23,25]. It is worth noting that the above policies existed in China before the implementation of MMT and there have been almost no new major public relations and marketing campaigns since the implementation of MMT. Therefore, we think the drop in HIV rates associated with injected drug use may be mainly attributable to MMT policies. The second is the reason why sexual behaviors rather than injected drug use increased as a proportion of HIV causes. Sexual behavior-induced HIV includes heterosexual and homosexual behaviors, and aspects of commercial sexual behavior, which is related to urbanization and population shifts among other processes [26,27]. Compared with HIV infections related to injected drug use, HIV infections related to commercial sexual behaviors may be more difficult to control, because commercial sexual behaviors occur within many kinds of entertainment venues, such as salons, bars, nightclubs, bath centers, and hotels. Condom providing and health education (especially the involving sexual workplace) are the major public policy being taken to address the rise in HIV cases cause by sexual behaviors.

### Problems identified in MMT treatment program in Hubei province, China

Although MMT effectively reduces drug abuse and prevents new HIV infections, there are two problems worth noting, namely the high drop-out rate and the poor information collection on and management of MMT clients. The current study used registration data from MMT clinics as no data from a specific survey of drop-outs were available. According to existing research on MMT in China, the reasons for dropping out of MMT—in addition to demographic factors such as gender, education level, and age—mainly include frequent contact with drug abusers, crime, being remanded in custody for drug abuse, and going to work in another city [28-31]. Based on our examinations of the existing research and the practice of MMT in Hubei Province, we think the reasons for client dropout may be related to social issues the clients encounter, not physiological or biological issues. Furthermore, although biological measures such as providing methadone and needle exchanges deal with the physiological withdrawal symptoms and biological virus infections, dropout follow-up and sustainable MMT sustainable development requires social measures, especially family cooperation and employment opportunities, which would help MMT clients return to society without discrimination.

The second problem is that poor information collection on and management of MMT clients was ignored easily at the beginning of MMT projects in many developing countries. Surveys of MMT clients are often ignored, mainly because of poor perceptions of the surveys, a lack of awareness of the role of information itself (here, this information is essentially evidence), or a lack of awareness of the role of the planning, design and implementation of information management. Without information and evidence accumulation, many policy implications would reflect low-level replication. Facing low coverage and a high drop-out rate, it is important to have data collection in different regions and in-depth analysis of factors influencing MMT. In many developing countries, barriers to information and evidence accumulation may not be information technology itself, but rather the lack of awareness, especially the value of this information and the planning, design and implementation of information management.

## Conclusions

Following the implementation of the MMT policy in Hubei province the proportion of HIV infection among drug abusers declined for five consecutive years. However, high drop-out rate and poor information management have been particularly problematic. Client drop-out from MMT programs may reflect social issues the clients encounter, and consequently, the incorporation of social measures, for example involving client families or improving employment possibilities, is required in order to develop sustainable MMT programs that help MMT clients return to society without discrimination.

## Competing interests

The authors declare that they have no competing interests.

## Authors’ contributions

JD and YL worked together to designed, conducted and drafted the manuscript. JD and LZ performed the literature search and data extraction. All authors read and approved the final manuscript.
